# Intraoperative diagnosis and successful management of acute coronary–subclavian steal syndrome during off-pump coronary artery bypass grafting: a case report

**DOI:** 10.3389/fcvm.2026.1766112

**Published:** 2026-05-15

**Authors:** Da Gong, Fuqiang Zhang, Jin Wang

**Affiliations:** Department of Cardiac Surgery, Peking University First Hospital, Beijing, China

**Keywords:** coronary-subclavian steal syndrome, graft reconstruction, intraoperative diagnosis, off-pump coronary artery bypass grafting, transit-time flow measurement

## Abstract

Coronary–subclavian steal syndrome (CSSS) is an uncommon but potentially serious complication following coronary artery bypass grafting (CABG). In the presence of significant proximal left subclavian artery stenosis, blood flow within the left internal mammary artery (LIMA) may reverse, leading to myocardial ischemia in the left anterior descending artery (LAD) territory. CSSS typically manifests months to years after CABG, whereas intraoperative onset during the procedure is exceedingly rare and very few cases previously documented. We report a patient who developed acute CSSS during off-pump CABG. Prompt intraoperative diagnosis was achieved using transit-time flow measurement (TTFM), and the condition was successfully managed by immediate LIMA graft reconstruction. This case highlights the critical role of intraoperative flow assessment and timely surgical decision-making in the management of rare but life-threatening complications during CABG.

## Introduction

The left internal mammary artery (LIMA) is the preferred conduit for left anterior descending artery revascularization in coronary artery bypass grafting (CABG). Significant left subclavian artery stenosis may impair LIMA inflow and cause coronary–subclavian steal syndrome (CSSS), a rare but serious complication that usually presents late after surgery (1, 2). Intraoperative CSSS is exceedingly uncommon and poses a diagnostic challenge due to its abrupt onset and nonspecific clinical manifestations. We report a rare case of acute intraoperative CSSS during off-pump CABG, which was promptly diagnosed using transit-time flow measurement (TTFM) and successfully treated with immediate LIMA graft reconstruction. This case underscores the importance of intraoperative flow assessment and rapid surgical decision-making in preventing catastrophic myocardial ischemia.

## Case

A 65-year-old man (height 179 cm, weight 77 kg) presented with a 5-year history of recurrent xiphoid discomfort, recently aggravated for 1 month. He was admitted with a diagnosis of unstable angina. Coronary angiography performed on hospital day 2 revealed 95% stenosis of the left main coronary artery and total occlusion of the right coronary artery. He was transferred to the Department of Cardiovascular Surgery and scheduled for urgent off-pump CABG the following day due to severe coronary disease and recurring symptoms.

During off-pump CABG, the LIMA–LAD anastomosis and a sequential saphenous vein graft (SVG) to the second obtuse marginal (OM2) and diagonal branches were completed uneventfully. While preparing for proximal SVG-to-aorta anastomosis, the patient developed refractory ventricular fibrillation. Multiple electrical defibrillations failed to restore sinus rhythm. Sustained internal cardiac massage was performed, and emergent cardiopulmonary bypass (CPB) was established via the ascending aorta and right atrium. The final proximal anastomosis was completed under CPB support.

**Figure 1:**
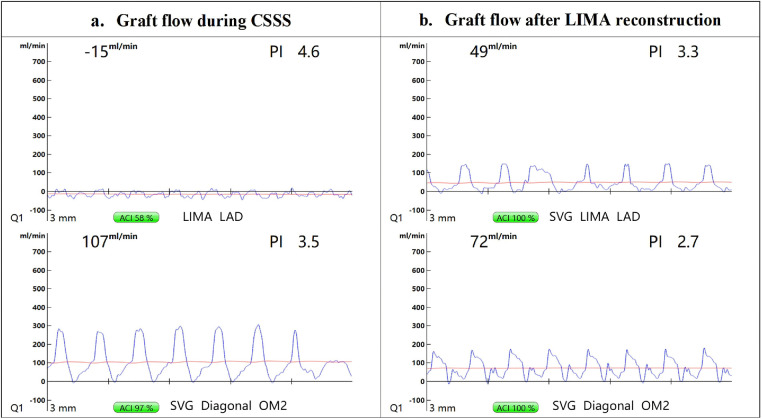
Transit-time flow measurement (TTFM). **(a)** During the episode of CSSS, TTFM showed retrograde flow in the LIMA–LAD graft (–15 mL/min, PI: 4.6), whereas the SVG-Diagonal-OM2 graft demonstrated antegrade flow (107 mL/min, PI: 3.5). **(b)** After LIMA reconstruction, repeat TTFM demonstrated restoration of antegrade flow in the LIMA–LAD graft (49 mL/min, PI: 3.3), and the flow in the SVG-Diagonal-OM2 graft remained satisfactory (72 mL/min, PI: 2.7). CSSS, coronary–subclavian steal syndrome; LIMA, left internal mammary artery; LAD, left anterior descending artery; SVG, saphenous vein graft; OM2, second obtuse marginal branch.

Due to intraoperative myocardial ischemia and depressed ventricular function, an intra-aortic balloon pump (IABP) was inserted via the right femoral artery. TTFM (Medistim MiraQ™) demonstrated persistent retrograde flow in the LIMA graft (–15 mL/min; PI 4.6) with normal antegrade SVG flow (107 mL/min; PI 3.5) (Figure 1a). Concurrently, a marked pressure difference was observed: IABP balloon tip pressure exceeded left radial arterial pressure by approximately 30–40 mmHg.

**Figure 2:**
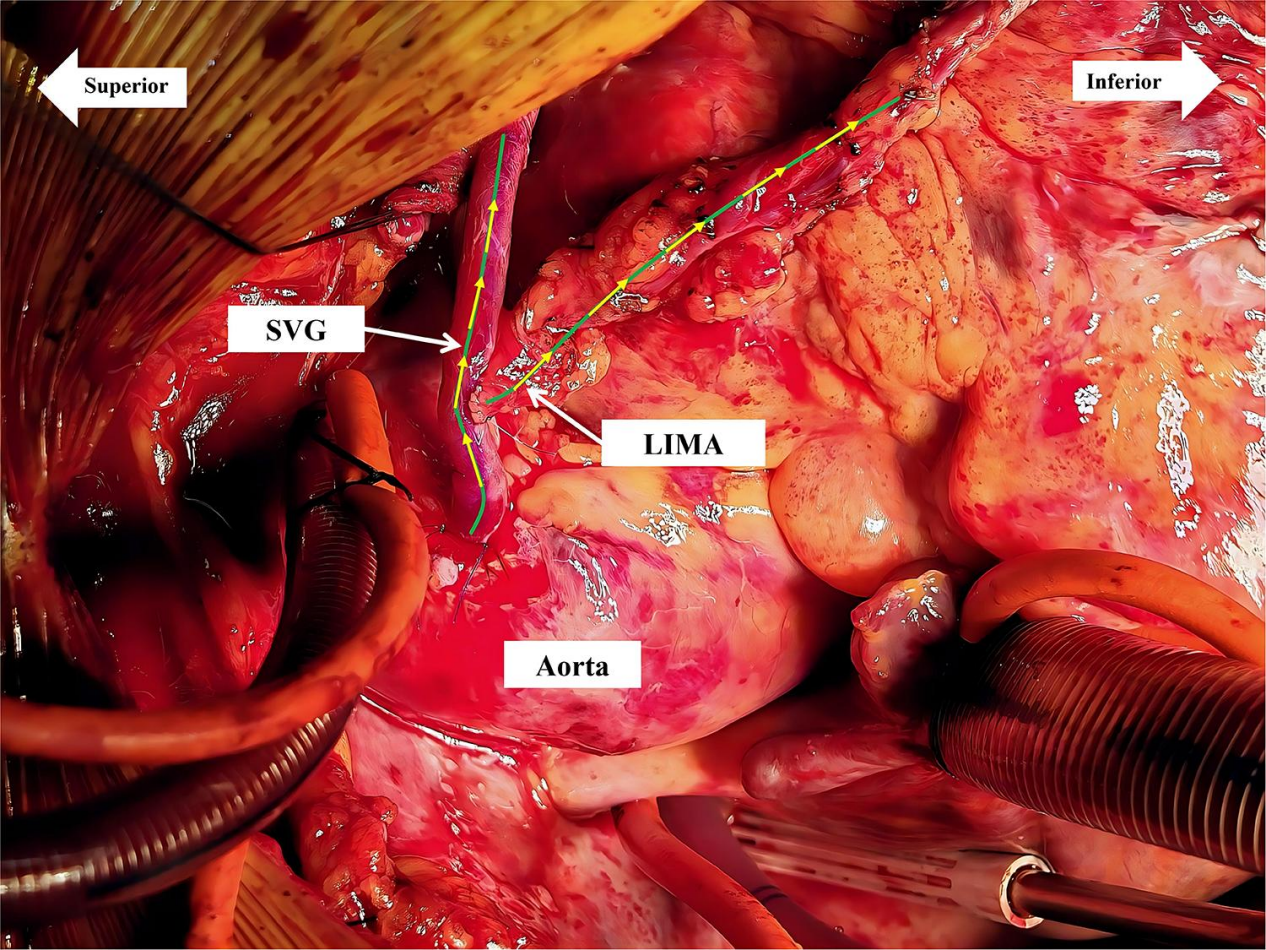
Y-shaped graft: the LIMA was divided and anastomosed to the proximal SVG. LAD, left anterior descending artery; SVG, saphenous vein graft.

Taken together, these findings strongly suggested critical left subclavian artery stenosis causing acute CSSS, which likely triggered the refractory ventricular fibrillation. The proximal LIMA was promptly divided and anastomosed to the proximal SVG as a Y-composite graft (Figure 2). Post-reconstruction TTFM confirmed restoration of antegrade LIMA flow (49 mL/min; PI 3.3) (Figure 1b).

With IABP support, the patient's hemodynamics gradually stabilized, and the patient was weaned from CPB. The procedure was maintained on a beating heart. CPB and operative times were 76 and 335 min, respectively.

**Figure 3:**
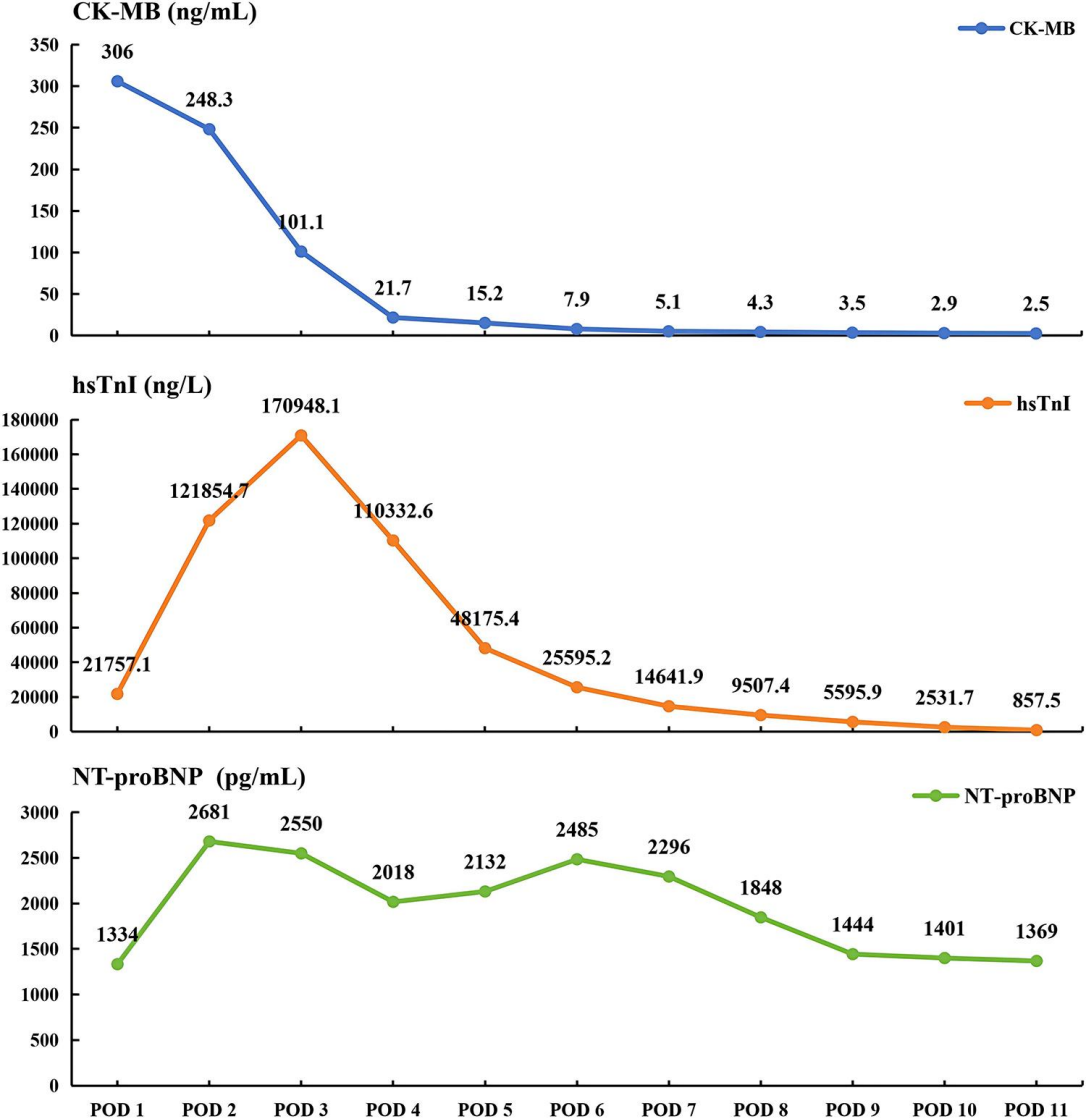
Trends in postoperative cardiac biomarkers (CK-MB, hsTnI, NT-proBNP). POD, postoperative day; CK-MB, creatine kinase-muscle brain isozyme; hsTnI, high-sensitivity Troponin I; NT-proBNP, N-terminal pro-B-type natriuretic peptide.

Postoperatively, the patient remained hemodynamically stable with no arrhythmia recurrence. Cardiac biomarkers peaked at CK-MB 306 ng/mL, hsTnI 170,948.1 ng/L, and NT-proBNP 2,681 pg/mL, followed by a steady decline to 2.5 ng/mL, 857.5 ng/L, and 1,369 pg/mL, respectively, by discharge (Figure 3). Compared to preoperative values (LVEF 52.3%, LVEDD 48 mm), echocardiography on postoperative day 10 showed an LVEF of 56% and an LVEDD of 41 mm, suggesting no significant alteration in overall cardiac function.

He was extubated 33.7 h after surgery, and IABP support was discontinued at 80.6 h. The ICU stay was 6.5 days, and total hospital stay was 16 days. Ultrasonography demonstrated approximately 80% stenosis of the left subclavian artery. No symptoms of vertebrobasilar insufficiency or left upper extremity ischemia occurred postoperatively. The patient is scheduled to undergo elective subclavian artery stenting 1 month after recovery from cardiac surgery.

### Discussion

**Figure 4:**
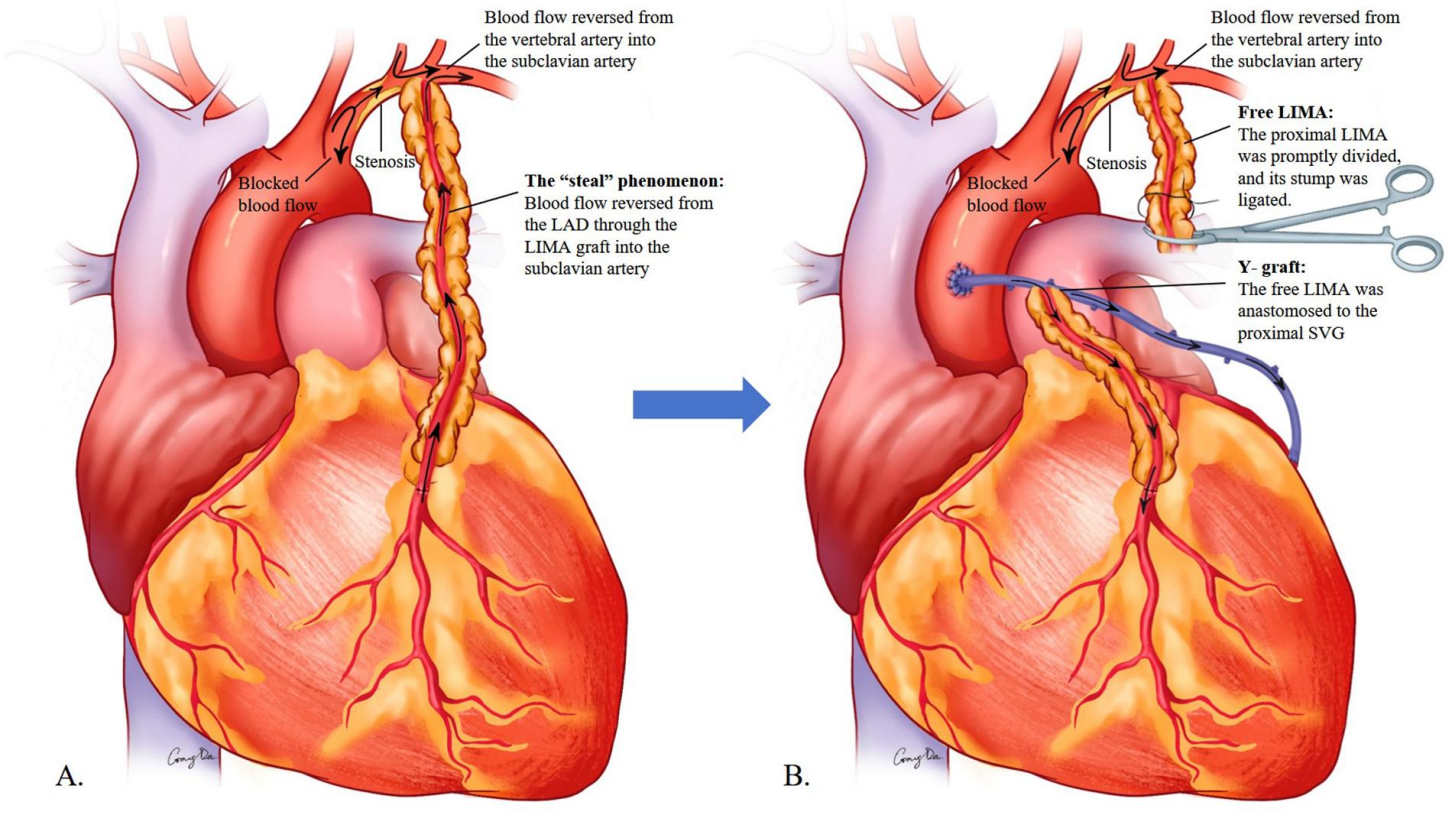
**(A)** Coronary–subclavian steal syndrome: the retrograde flow from the LAD via the LIMA to the subclavian artery. **(B)** A composite Y-graft was constructed by the free LIMA end-to-side to a SVG. LAD, left anterior descending artery; LIMA, left internal mammary artery.

CSSS represents a clinically important but often underrecognized complication in CABG patients with unappreciated proximal left subclavian artery stenosis (Figure 4A). The modern concept of “pan-vascular disease” emphasizes that atherosclerosis is a systemic process frequently affecting multiple vascular territories. Epidemiologic studies have shown that 2.3%–6.8% of patients with coronary artery disease also exhibit subclavian artery stenosis, with lesions more commonly affecting the left side (3–7). Although the LIMA remains the preferred conduit for LAD revascularization because of its superior long-term patency and excellent target-vessel compatibility, significant subclavian stenosis can critically compromise graft inflow and lead to CSSS.

In elective CABG, routine preoperative evaluation—particularly ultrasonography of the supra-aortic branches—facilitates early detection of subclavian disease. In urgent or emergent cases, however, the time available for full vascular assessment is often limited. Under these circumstances, simple bedside indicators such as an inter-arm systolic blood pressure difference greater than 15 mmHg provide a practical and valuable screening method (4, 8, 9). When subclavian disease cannot be confidently excluded, alternative conduits such as the right internal mammary artery or saphenous vein graft should be considered to avoid compromised flow through the LIMA.

Intraoperative evaluation revealed a 30–40 mmHg gradient between the IABP and left radial artery pressures. At our institution, bilateral radial artery monitoring during CABG is not routinely performed unless specifically indicated. Prior to the onset of CSSS, hemodynamic management was guided exclusively by left radial artery monitoring, which likely underestimated true central aortic pressure and predisposed to circulatory misinterpretation. Consequently, the intraoperative ventricular fibrillation might be attributable not only to CSSS but also to suboptimal hemodynamic management secondary to unilateral monitoring.

Furthermore, preoperative subclavian angiography was omitted due to clinical urgency and our standard right transradial approach, which does not routinely incorporate concomitant subclavian imaging. Acknowledging this limitation, future protocols will integrate subclavian angiography during initial coronary angiography for high-risk patients presenting with significant inter-arm pressure discrepancies. Therefore, we advocate for routine preoperative bilateral upper-limb blood pressure assessment in patients undergoing emergency CABG. Should a significant inter-arm discrepancy be identified, simultaneous bilateral upper-limb or combined upper- and lower-limb arterial monitoring is recommended to ensure hemodynamic accuracy and enhance procedural safety.

The present case demonstrates the critical role of intraoperative physiological assessment in diagnosing acute CSSS. TTFM provided unequivocal evidence of retrograde flow within the LIMA–LAD graft, directly establishing the presence of a steal phenomenon. Although graft TTFM currently carries a Class IIa, Level B recommendation, its importance is magnified in high-risk, unstable, or emergency CABG, where rapid identification of graft dysfunction is essential (10).

Management of acute CSSS requires prompt recognition and decisive intervention. In cases with hemodynamic collapse or malignant arrhythmias, rapid initiation of CPB remains the safest and most reliable method of restoring systemic and coronary circulation. Definitive correction involves reconstructing the compromised graft inflow—achieved here by creating a composite Y-graft with an SVG, though other options include direct re-anastomosis of the LIMA to the ascending aorta or substitution with alternative conduits (Figure 4B). A similar Y-graft strategy was employed by Saito et al. in a hemodialysis patient who developed intraoperative CSSS secondary to an ipsilateral arteriovenous fistula, further supporting the efficacy of this salvage technique (11).

Mechanical circulatory support such as IABP or venoarterial extracorporeal membrane oxygenation (VA-ECMO) may be required for patients with severely impaired ventricular function. As illustrated in this case, timely surgical correction coupled with adequate circulatory support can prevent irreversible myocardial injury and allow for full recovery.

Regarding the limitations of this study, intraoperative measurement of LIMA free flow was not performed due to the critical condition, characterized by impending circulatory collapse and severe myocardial ischemia, necessitating immediate intervention. Had cardiopulmonary bypass been established and hemodynamics stabilized, assessment of LIMA free flow might have helped identify potential graft-related issues and improved diagnostic confidence. In addition, TTFM did not include the Diastolic Fraction (DF). Owing to the lack of a compatible ECG connection cable for the Medistim MiraQ™ cardiac flowmeter at our center, DF could not be recorded intraoperatively. Inclusion of DF might have further enhanced the diagnostic value and reliability of the flow assessment.

## Conclusion

In urgent or emergent CABG, subclavian artery stenosis may be overlooked because of the limited time available for comprehensive preoperative evaluation. This case emphasizes several key principles for preventing and managing CSSS. Bilateral upper-limb blood pressure measurement serves as a simple yet valuable screening tool for detecting possible subclavian disease. Intraoperative graft flow measurement provides crucial real-time physiologic data and enables early detection of CSSS. Finally, prompt diagnosis and rapid graft reconstruction, supported when necessary by mechanical circulatory devices, are essential for achieving favorable outcomes. This case contributes meaningful insights into the prevention, diagnosis, and management of CSSS during both off-pump and on-pump CABG procedures. Consequently, routine TTFM should be recommended in all CABG cases where internal mammary artery is used, especially in urgent settings.

## Data Availability

The original contributions presented in the study are included in the article/Supplementary Material, further inquiries can be directed to the corresponding author.
